# Use of Nanomaterial-Based (Micro)Extraction Techniques for the Determination of Cosmetic-Related Compounds

**DOI:** 10.3390/molecules25112586

**Published:** 2020-06-02

**Authors:** José Grau, Juan L. Benedé, Alberto Chisvert

**Affiliations:** Department of Analytical Chemistry, University of Valencia, Burjassot, 46100 Valencia, Spain; jose.grau-escribano@uv.es (J.G.); benede@uv.es (J.L.B.)

**Keywords:** cosmetic-related compounds, microextraction techniques, nanomaterials, sample preparation

## Abstract

The high consumer demand for cosmetic products has caused the authorities and the industry to require rigorous analytical controls to assure their safety and efficacy. Thus, the determination of prohibited compounds that could be present at trace level due to unintended causes is increasingly important. Furthermore, some cosmetic ingredients can be percutaneously absorbed, further metabolized and eventually excreted or bioaccumulated. Either the parent compound and/or their metabolites can cause adverse health effects even at trace level. Moreover, due to the increasing use of cosmetics, some of their ingredients have reached the environment, where they are accumulated causing harmful effects in the flora and fauna at trace levels. To this regard, the development of sensitive analytical methods to determine these cosmetic-related compounds either for cosmetic control, for percutaneous absorption studies or for environmental surveillance monitoring is of high interest. In this sense, (micro)extraction techniques based on nanomaterials as extraction phase have attracted attention during the last years, since they allow to reach the desired selectivity. The aim of this review is to provide a compilation of those nanomaterial-based (micro)extraction techniques for the determination of cosmetic-related compounds in cosmetic, biological and/or environmental samples spanning from the first attempt in 2010 to the present.

## 1. Introduction

The growing social concern about beauty has encouraged in last decades a remarkable increase in the use of cosmetic products. These products are used daily by many consumers, contributing to the improvement of their well-being. To ensure their safety, these products are regulated worldwide, so that the different regulations in force in each country prohibit and restrict (in terms of concentration, type of product, users, etc.) the use of certain compounds [1]. In this sense, analytical methods to perform the control of cosmetic products, not only to monitor the prohibited substances but also the allowed ingredients, to ensure their efficacy are demanded by authorities and by the cosmetic industry itself [2].

It is important to note that, given the high responsibility of the cosmetic industry, the presence of prohibited substances in the cosmetic products as consequence of their intentional use is not expected. Therefore, their presence at trace level could be due to unintended causes (e.g., impurities from raw materials, degradation of some ingredients, migration of compounds from the containers, or even undesired reactions between cosmetic ingredients during the manufacturing or storage processes). 

Moreover, different studies have shown that after the application of a cosmetic product, some of its ingredients might be percutaneously absorbed into the organism [3]. Then, they are distributed throughout the organism by blood where they can be altered producing different metabolites that might cause adverse effects on health [4,5,6] due to their endocrine disrupting and/or carcinogenic properties [7]. To this regard, analytical methods are needed to carry out studies of percutaneous absorption, metabolism and/or excretion of these cosmetic ingredients and their metabolites.

Likewise, due to the increasing use of cosmetic products, some of their ingredients have reached the environment by direct and indirect sources, and they are being accumulated in surface waters or sediments, having a negative effect on the different ecosystems even at trace levels [8,9,10]. Here again, analytical methods are needed to allow the environmental surveillance of cosmetic ingredients.

For all the above reasons, it has been necessary to develop analytical methods of high selectivity and sensitivity for the determination of traces of these cosmetic-related compounds in these three scenarios: cosmetic, biological and environmental matrices. 

In this regard, extraction techniques are required for enrichment purposes. Traditional liquid–liquid extraction (LLE) and solid-phase extraction (SPE) are time-consuming and use high quantities of organic solvents. Thus, the employment of the so-called microextraction techniques have been considered as better alternatives because they not only reduce the use of solvents and extraction times but also allow to obtain lower limits of detection (LODs).

On this matter, sorbent-based microextraction techniques have a huge impact nowadays. In these techniques, in the first step, the analytes are adsorbed by the extractant phase. Subsequently, compounds are selectively desorbed into a small amount of solvent (liquid desorption) or introduced directly in the GC system for thermal desorption (TD).

Among the sorbent-based microextraction techniques, those based on the use of nanomaterials as extraction phase have attracted attention during the last years. Their higher surface area, compared with macroscopic materials, and their easy surface modification, which allows to synthesize a great diversity of superficially modified sorbents and thus to increase their selectivity with regard to the target analytes [11], make them interesting alternatives for sorbent-based microextraction techniques.

This review presents a comprehensive compilation of those published papers on the application of nanomaterial-based (micro)extraction techniques to the determination of cosmetic-related compounds in different matrices, such as cosmetic products, biological and environmental samples, spanning from the first attempt in 2010 to the present.

## 2. (Nano)Materials in Sorbent-Based Microextraction Approaches

In sorbent-based extraction approaches, sorbents play a crucial role to get selective, precise and accurate enrichment of the analytes. Several sorbents with different compositions and physicochemical properties have been used. Moreover, combination of different materials, including nanometric and micrometric materials, gives rise to hybrid nanomaterials or composites. An important feature of these resulting sorbents is that they maintain the properties of both original materials. Some of those materials used in microextraction techniques are briefly presented below.

Regarding to nanometric materials, one of the most popular materials are the nanoparticles (NPs). NPs are small spheres between 1 and 100 nm of a wide range of metallic and metallic oxide materials. In this group, NPs of noble metals (i.e., AuNPs and AgNPs) [12] are commonly used due to their chemical stability, elevated adsorption and high ratio surface/volume. Most recently, magnetic NPs (MNPs) have gained a considerably interest. They present similar properties to nonmagnetic NPs, such as high surface and huge adsorption capacity, but their main advantage compared with the nonmagnetic ones is the easy retrieval after the extraction, since only an external magnetic field is necessary [13]. Moreover, the MNPs can be easily coated with other materials maintaining the nanometric size or can be embedded on the surface of the material to obtain magnetic composites [14]. Traditionally, ferrite NPs (Fe_3_O_4_) have been preferred, but its low stability and its facility to form aggregates make a coating step (e.g., with silica shell) necessary to protect them from oxidation. On the other side, cobalt ferrite MNPs (CoFe_2_O_4_) have proved to be more stable, and no additional steps are needed in order to protect them [15].

Carbonaceous nanomaterials such as carbon nanotubes (CNTs), either single-walled (SWCNTs) or multiwalled (MWCNTs), graphene oxide (GO), reduced graphene oxide (rGO) or carbon dots (CDs) are also widely used as sorbents. MWCNTs can be used as sorbent themselves due to their high surface area and their ability to have hydrophobic, π-π and/or electrostatic interactions [16] or can be used to create composite materials maximizing the specific surface area of the original sorbent material [17]. Graphene derivatives (GO or rGO) are preferred than graphene mainly for economic reasons. GO is obtained by the oxidation of graphite, whereas rGO is prepared by the reduction of GO. GO is preferred to analyse polar compounds since its surface has polar groups (i.e., alcoholic, carboxyl and epoxy groups). In contrast, when the reduction is produced to obtain rGO, most of these groups disappear, which makes rGO an ideal sorbent for non-polar analytes. Both GO and rGO show high surface area and thermal and chemical stability that make them really efficient sorbents [18]. Finally, CDs are nanoparticles that possess unique optical properties similar to the well-known quantum dots, but they are safer and less harmful for the environment [19].

Metal–organic frameworks (MOFs) are nanomaterials recently employed as sorbents in microextraction approaches. They are three-dimensional inorganic–organic crystalline structures formed by the assembly of metal ions and organic ligands by coordinative bonds or different polymers with different interactions for the extraction of different analytes. Their properties vary depending on the ligands used and/or their geometry. They present interesting properties to be used as sorbents, such as high chemical and thermal stability, large porosity and huge surface area. In fact, the high stability allows some of them to be reused more than 100 times [20]. On the other hand, covalent organic frameworks (COFs), most recently used as sorbents in extraction techniques, consist in the assembly between different units by covalent bonds, and their structures may adopt a two- to three-dimensional form depending on the application. Similar to MOFs, they have large surface area, high chemical stability and high porosity. Furthermore, they present other properties such as low density and tunable pore size and structure [21,22].

Finally, layered double hydroxides (LDH) are two-dimensional nanosorbents composed by two layers of divalent and trivalent cations with an anionic interphase. The anions in the interlayer can be easily exchanged by other anions [23] and, for that reason, they are normally employed for the determination of anionic compounds.

Along with nanomaterials, non-nanometric sorbents are usually employed to enhance the selectivity and the extraction capability. 

In this regard, polymers are micrometric structures synthetized either from the same type of monomer or employing two or more types of monomers (copolymerization) [24]. Different polymers have been widely used as sorbents due to their good extraction properties. Moreover, composite materials made of polymers combined with NPs present higher porosity when compared to the naked polymers [16]. 

When copolymerization of functional monomers and a cross-linker is performed in the presence of a template molecule, the so-called molecularly imprinted polymers (MIPs) are obtained. The cavities formed by this template allow to have a very selective sorbent, since they are complementary in size, shape and chemical environment to the analyte. MIPs can be synthetized for just one analyte, if only one template is used or can be prepared with multiple templates to recognize different analytes, enhancing its versatility [25]. 

Finally, ionic liquids (ILs), which are melt salts at temperature below 100 °C made of a combination between organic cations and different inorganic or organic anions, have been widely used in analytical methods due to their interesting properties, such as high extractability, elevated thermal stability and negligible vapor pressure. Besides those mentioned before, their viscosity and miscibility can be modified for specific applications [26,27]. 

## 3. Nanomaterials-Based Microextraction Approaches Used for the Determination of Cosmetic-Related Compounds

**Figure 1:**
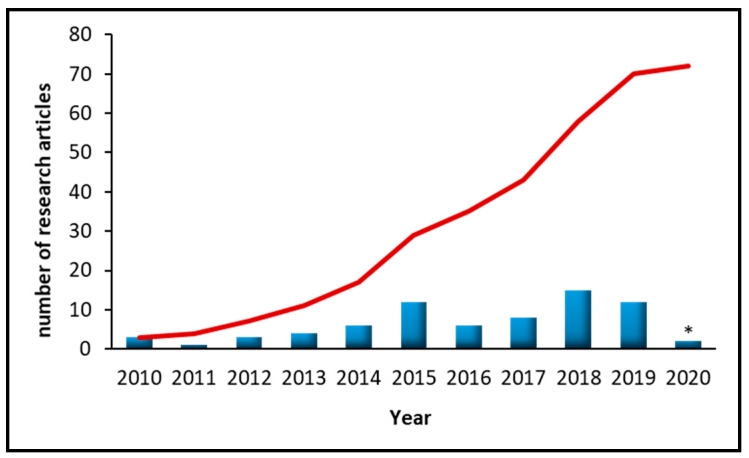
Number of research articles published in the last 10 years about the use of nanomaterial-based (micro)extraction techniques for the determination of cosmetic-related compounds (red line represents the accumulated number; * current year).

In this review, those published articles employing nanomaterials for the extraction (or determination) of cosmetic-related compounds in cosmetic, biological or/and environmental samples are compiled and briefly discussed. From the first one in 2010 up to the present, more than 70 articles have been published, with a clear increase every year, representing a trend within the analytical chemistry field. Figure 1 shows a histogram of all these research articles according to year of publication.

### 3.1. Solid Phase Extraction 

Briefly, classical solid-phase extraction (SPE) process consists of percolating the sample solution through a cartridge (or disc) containing the solid sorbent that retains the target analytes, whereas the rest of the sample is discarded. After a cleaning step, an elution solvent is passed to desorb and to retrieve the analytes.

As it can be seen in Table 1, different nanomaterials have been packed in SPE-cartridges for the determination of cosmetic-related compounds. In this sense, Márquez-Sillero et al. [28] employed MWCNTs for the determination of four parabens in cosmetic products, previously lixiviated with water. Wang et al. [29] developed a GO sponge for the determination of six benzotriazole compounds in sewage and cosmetic samples. The use of MIPs in SPE for cosmetic analysis was first proposed by Zhu et al. [30], who used MIP-coated silica nanoparticles for the determination of bisphenol A (BPA) in shampoos and bath lotions, which were previously lixiviated with toluene before introducing them into the cartridge. Later, Wang et al. [31] functionalized MWCNT with a prednisone-template MIP for the determination of this glucocorticoid. Zhong et al. [32] employed carboxylated GO with polyvinyl chloride (PVC) as sorbent to determine different sulphonamides as contaminants in cosmetics products, and Abdolmohammad-Zadeh et al. [33] created a LDH cartridge with nickel and zinc for the analysis of p-aminobenzoic acid in cosmetic samples, which was dissolved in a proper water or ethanol amount before the extraction. 

It should be noticed that SPE is not in fact a microextraction technique, but the use of nanomaterials as sorbents allows to achieve low LODs (from ng mL^−1^ to ng L^−1^), which are suitable for the trace analysis of cosmetic-related compounds in the different matrices considered.

### 3.2. Solid Phase Microextraction

Solid phase microextraction (SPME) was developed by Arthur and Pawliszyn in 1990 [34]. In this technique, analytes are retained on a fibre coated with the sorbent material. The extraction can be performed by direct immersion into the sample or, if the analytes are volatile enough, by setting the fibre in the head space. After the extraction, analytes are, usually, thermally desorbed, although in a minor extent, liquid desorption in an appropriate solvent has also been used. Several methods based on SPME have been employed for the extraction of cosmetic-related compounds from different matrices. They are all listed in Table 2.

With that aim, different works for determination of parabens in different matrices have been reported. Ara et al. [35] modified mesoporous silica nanoparticles with polyaniline (PANI) and p-toluene sulphonic acid to coat the fibre for the determination of three of these target compounds in various cosmetics creams and wastewater. Yazdi et al. [36] determined the same parabens in wastewater samples employing AgNPs embedded on polypyrrole. First of all, pyrrole was polymerized on the hollow fibre, and then, it was introduced in a suspension of AgNPs for bounding. 

For the determination of UV filters in environmental samples, different titanium oxide-based fibres have been used due to their excellent properties, such as high chemical and thermal stability, low cost and toxicity and good biocompatibility. In this sense, Du and coworkers used PANI-coated titania nanotubes (NTs) [37], ZrO_2_-based fibre [38] and TiO_2_ NPs functionalized with phenyl groups [39] for the analysis of different UV filters in river water and wastewater. The same authors also used electrodeposited AuNPs onto a stainless-steel wire followed by a coating step with 1,8-octanedithiol [40] for the same purpose. Moreover, Mei et al. [41] synthesized a polymeric ionic liquid (PIL) with MNPs to enhance the extraction capability of diamagnetic UV filters employing magnetic field gradients. This method was applied to lake and river waters and wastewater.

In addition to parabens and UV filters, extraction of other cosmetic-related compounds has been also performed by SPME. Wu et al. [42] employed a graphitic carbon nitride (g-C_3_N_4_) modified with rGO for the analysis of six polycyclic aromatic hydrocarbons (PAHs) in cosmetic products previously diluted in water. Tong et al. [43] synthesized a polymeric monolith by copolymerization of butyl methacrylate (BMA) and ethylene dimethacrylate (EDMA), followed by the addition of rGO nanosheets for the analysis of nine glucocorticoids (GCCs). In this methodology, GCCs were first extracted with acetonitrile (ACN) and then, SPME was performed. Finally, Wang et al. [44] used hydroxyapatite (HAP) NPs to coat a titanium fibre for the analysis of different chlorophenols, BPA and triclosan (TCS) in river water and sewage.

All the analyses reported with SPME show a great sensitivity, proving to be one of the most appropriate techniques for the analysis of traces. As shown in Table 2, the lowest LODs are achieved for those methods focused on the analysis of environmental samples (mostly waters). Since cosmetic matrices are usually difficult matrices, a clean-up step with organic solvents is usually required, which reduces the sensitivity of the method due to the dilution effect, but in any case, the achieved LODs are low enough to analyse the cosmetic samples. 

### 3.3. Stir Bar Sorptive Extraction 

Stir bar sorptive extraction (SBSE), introduced at the end of the 1990s by Baltussen et al. [45], consists on a stir bar coated with the extractant material. This functionalized stir bar is then introduced in the sample and stirred in order to extract the analytes. As it is shown in Table 3, just four articles employing nanomaterials-coated stir bars have been reported. Wang et al. [46] immobilized MIL-68 MOF onto the stir bar surface for the analysis of three parabens from pretreated sunscreen and plasma samples. Fresco-Cala et al. [47] developed a hybrid monolith composed by carbon nanohorns and a polymer formed by methacrylate monomers for the analysis of five benzophenone-type UV filters in urine and water samples. Siritham et al. [48] extracted butylated hydroxytoluene (BHT), butylated hydroxyanisole (BHA) and other antioxidants from different cosmetic products, such as conditioners, hair shampoos and mouthwash. First, samples were treated due to their high viscosity. Then, a composite based on GO, polyethylene glycol and natural latex was added for the microextraction procedure. Finally, Zang et al. [49] determined four chlorophenols employing a stir bar fabricated by filling a hollow tube with a Fe_3_O_4_-rGO-g-C_3_N_4_ composite. 

Similar to SPME, excellent LODs are achieved by SBSE for all those analysed matrices. However, despite the simplicity of this technique, the necessity of higher extraction times, sometimes more than 2 h, makes it less attractive for this application, and other techniques based on the dispersion of the sorbent, as discussed below, are preferred.

### 3.4. Dispersive Solid Phase Extraction 

Dispersive solid phase extraction (DSPE) has become a widely used extraction technique since its proposal by Anastassiades et al. in 2003 [50]. Traditionally, the sorbent is introduced and dispersed into the sample. When the extraction is completed, the sorbent is recovered by means of centrifugation and decantation. However, nowadays, this technique has gained more interest due to the introduction of magnetic materials as sorbents, allowing an easy recovery of the sorbent by employing an external magnetic field, which considerably reduces the analysis time.

As can be seen in Table 4, 38 articles employing DSPE for the determination of cosmetic-related compounds have been reported, and only 6 of them resort to nonmagnetic sorbents, which shows the high impact that magnetic materials have caused in this extraction technique. In this sense, Rocío-Bautista et al. [51] used the MOF HKUST-1 in vortex-assisted DSPE for the extraction of a group of seven parabens in cosmetic creams, urine and environmental waters. Rashvand et al. [52] also analysed two parabens in wastewater samples by employing a GO-PANI composite. Li et al. [53] dispersed the MOF MIL-101 (Cr) in toner samples for the determination of different benzophenones. Gao et al. [54] synthesized a TCS-based MIP on CNTs in order to extract this analyte from lake and river waters. Zhai et al. [55] developed a method for the determination of hormones employing the MOF MIL-101 that was dispersed into the cosmetic sample after its dilution in a saline solution. Finally, Liu et al. [56] achieved the extraction of Hg(II) from cosmetic samples by measuring the fluorescence of CDs obtained from grass carps after their interaction with the analyte. 

On the other hand, several magnetic composites have been reported, especially focused on the study of parabens and TCS in different matrixes and UV filters in environmental samples. With that aim, Tahmasebi et al. [57] used PANI-coated Fe_3_O_4_ MNPs for the determination of three parabens in wastewaters, cosmetic creams and toothpaste. Ghambari et al. [58] employed recycled polystyrene (PS) to synthesize a composite with CoFe_2_O_4_ MNPs to determine a group of four parabens in river, creek and tap waters by using vortex to disperse the composite. Abbasghorbani et al. [59] used a magnetic composite of aminopropyl (AP) and Fe_3_O_4_ MNPs for the determination of five parabens in different aqueous samples. Ariffin et al. functionalized the Fe_3_O_4_ with different surfactants, such as Sylgard 309 [60] and DC193C [61], for the extraction of different parabens in lake, river and sea waters. Casado-Carmona et al. [62] created a hybrid material based on MNPs and an IL (i.e., MIMPF_6_) for the determination of four parabens along with some benzophenones and BPA in pool waters. The extraction was performed by dispersing the Fe_3_O_4_@MIMPF_6_ by ultrasounds and employing vortex agitation to achieve the adsorption of the analytes. Mehdinia et al. [63] immobilized self-doped PANI on a Fe_3_O_4_-rGO composite for the determination of various parabens in different cosmetics (sunscreen, toothpaste and moisturizing cream) pretreated with MeOH. Later, the same authors [64] compared different silica-based magnetic nanocomposites for the extraction of parabens from various cosmetic samples. Feng et al. [65] also worked with rGO for determination of two parabens in cosmetic samples. In this case, Fe_3_O_4_ MNPs were embedded into the rGO surface, and then, it was covered by layers of mesoporous silica (mSiO_2_) with phenyl-functionalized pore walls. Ultrasounds were employed for the dispersion of the material. 

Jalilian et al. [66] modified the MOF MIL-101 surface with Fe_3_O_4_ MNPs and MWCNTs for the determination of two parabens along with three phthalates in both cosmetic creams and tap water. Cosmetic products were previously dissolved in MeOH:H_2_O before the extraction. The use of a COF as sorbent was proposed by Shavar et al. [67], who functionalized Fe_3_O_4_ MNPs with a covalent triazine-based COF for the determination of a group of four parabens in water, cosmetic products and breastmilk. Yusoff et al. [68] synthesized a magnetic composite with Fe_3_O_4_ MNPs coated with the IL 1-butyl-3-methylimidazolium chloride. This sorbent was applied to the extraction of four parabens in river, pond and lake waters and in MeOH pretreated cosmetic creams. Pastor-Belda et al. [69] precipitated Fe_3_O_4_ MNPs on MWCNTs surface for the analysis of several parabens in water and urine. Ghasemi et al. [70] employed γ-Fe_2_O_3_ MNPs coated with HAP to determine six parabens in soils, water and urine assisted by ultrasounds. Before DSPE procedure, soil samples were lixiviated in water.

Regarding the analysis of UV filters in environmental samples, Wang et al. [71] performed the extraction of three benzophenones in soils with Fe_3_O_4_ MNPs combined with MOF-1210 (Zr/Cu). Piovesana et al. [72] employed graphitized carbon black (GCB) prepared with MNPs for the extraction of 10 UV filters in different surface waters. Cheng et al. [73] used polydopamine-coated Fe_3_O_4_ MNPs for the analysis of 11 UV filters in wastewaters. Román-Falcó et al. [74] covered the CoFe_2_O_4_ MNPs surface with oleic acid. The extraction and subsequent determination of six UV filters was accomplished in tap, river and sea waters. Giokas et al. [75] developed a method for the determination of four UV filters, consisting a cloud-point (CP) extraction followed by a DSPE step in the micellar phase using core–shell Fe_2_O_3_@C coated with polysiloxane (PSx).

Regarding to TCS determination, Yang et al. [76] performed the microextraction in toothpastes previously lixiviated in MeOH. For the DSPE step, MIL-101 MOF was functionalized with Fe_3_O_4_. Li et al. [77] analysed TCS and triclocarban (TCC) in biological samples employing a magnetic COF formed by the condensation of 1,3,5-tris(4-aminophenyl) benzene (TAPB) and terephthaldicarboxaldehyde (TPA) on the surface of the MNPs. Li et al. [78] employed GO embedded with magnetic iron nanowires for the analysis of TCS in lake water and wastewater along with BPA, and Jiang et al. [79] synthesized a Fe_3_O_4_-PANI composite for the extraction of TCS, BPA and 2,4-dichlorophenol from water samples.

Besides those compounds mentioned before, other analytes have been also determined using nanomaterials. Three works have been reported on the analysis of GCCs in cosmetic products. Du et al. [80] employed Fe_3_O_4_ coated with a MIP for the determination of dexamethasone in skincare products. Liu et al. [81] prepared a magnetic composite based on MNPs coated with a dual template MIP for the determination of hydrocortisone and dexamethasone from different cosmetic products (lotions, masks and toners), which were previously treated with a saturated NaCl solution and acetonitrile (ACN). Finally, Li et al. [82] determined five GCCs in facial masks previously sonicated in ultrapure water, employing magnetically functionalized g-C_3_N_4_ bonded to MIL-101 MOF.

Moreover, the determination of the dye rhodamine B in different matrices has been also performed. In this regard, Khani et al. [83] worked with γ-Fe_2_O_3_ MNPs coated with imino-pyridine on hand washing soaps. Before the DSPE step, the samples were dissolved in water. Bagheri et al. [84] used Fe_3_O_4_ MNPs functionalized with poly(aniline-naphthylamide) (PAN) for its determination in shampoos, eye shadows and hand washing products.

Tarigh et al. [85] worked with lipstick samples for the determination of lead and manganese employing a composite of Fe_3_O_4_ MNPs and MWCNT. Before the extraction, samples were mineralized at 450 °C, and subsequently, the ashes were dissolved with nitric acid. Xia et al. [86] determined whitening agents working with Fe_3_O_4_ MNPs coated with a polymeric COF based on benzidine and 1,3,5-triformylphloroglucinol. Liu et al. [87] synthesized a MIP-coated Fe_3_O_4_ MNPs for the determination of metronidazole in cosmetic creams, lotions and powders, previously lixiviated with MeOH. Finally, Maidatsi et al. [88] prepared a magnetic composite of Fe_3_O_4_ MNPs and rGO functionalized with octylamine to determine different musks, allergens and phthalates in water samples. More recently, Zhang et al. [89] employed halloysite nanotubes (HNTs) that where first filled with CoFe_2_O_4_ MNPs and later assembled with Au-NPs on its surface using APTES. This composite was applied for the determination of 4,4′–thioaniline in hair dyes.

As described in Table 4, LODs between μg mL^−1^ and ng L^−1^ are achieved in DSPE-based methods, although as expected, the instrumental technique has a huge impact on this parameter. In this sense, despite LC-UV has been extensively used, it might be not enough sensitive for the determination of trace levels of some of the cosmetic-related compounds. For this reason, other options, such as LC-MS/MS, have been preferred.

Extraction times are similar regardless of the use of magnetic materials or not. However, the use of the magnetic ones avoids centrifugation steps to recover the sorbent in the extraction and desorption steps, which redounds in the reduction of the total time of analysis. 

Compared with other techniques, DSPE combined with nanosorbents allows excellent LODs, many times comparable with SPME and SBSE, but with the advantage of shortest extraction times, usually under 20 min.

### 3.5. Stir Bar Sorptive-Dispersive Microextraction 

A hybrid approach combining DSPE and SBSE, termed stir bar sorptive-dispersive microextraction (SBSDME), was introduced in 2014 by Benedé et al. [90]. In this technique, a magnetic sorbent coats the stir bar by means of magnetic interactions. When the stirring rate is high enough, the sorbent is dispersed in the sample until the stirring is stopped; at that moment, the magnetic composite containing the analytes is retrieved by the stir bar. This approach has been also employed for the determination of cosmetic-related compounds in different matrices (Table 5). Benedé et al. developed different strategies for determination of UV filters in environmental samples employing CoFe_2_O_4_ MNPs coated with oleic acid for the analysis of eight hydrophobic UV filters in environmental samples [90,91,92]. Later, the same authors developed a method based on CoFe_2_O_4_ MNPs embedded on nylon-6 polymer for the determination of six hydrophilic UV filters [93].

Recently, SBSDME has been applied for the determination of other types of analytes in different matrices. Grau et al. [94] applied this technique for the study of triphenyl phosphate (TPP) and its metabolite, diphenyl phosphate (DPP), in urine samples by means of CoFe_2_O_4_ incrusted onto a weak anion exchanger (Strata X-AW). Miralles et al. [95] functionalized MIL-101 MOF with CoFe_2_O_4_ for the determination of eight N-nitrosamines in cosmetic products. In this methodology, N-nitrosamines were first pre-extracted in hexane and then preconcentrated with SBSDME. Finally, Vállez-Gomis et al. [96] determined 10 PAHs in cosmetic creams employing rGO covered with CoFe_2_O_4_ MNPs, where samples were previously extracted with hexane, and then, SBSDME was performed on the hexane solution.

As can be seen in Table 5, similar LODs are obtained for SBSDME and DSPE with comparable extraction times. The main difference between these two approaches relies where the magnet is positioned, i.e., outside the solution in DSPE and inside the solution in SBSDME; thus, this last one does not require an external magnet to retrieve the sorbent. This fact alleviates losses of the extractant material in the different steps due to the reduction of sorbent and sample manipulation.

### 3.6. Other Sorbent-Based Microextraction Approaches 

Besides the most used microextraction techniques described above, other extraction approaches using nanomaterials for the determination of cosmetic-related compounds have been published. These methods are summarized in Table 6. Makkliang et al. [97] proposed rotative SPME using a multistir-rod microextractor based on MWCNT functionalized with carboxyl groups, which was applied for parabens determination in cosmetic samples previously dissolved in MeOH. Alcudia-León et al. [98] also determined parabens in pool and sea waters by magnetically confined hydrophobic nanoparticles microextraction. In this method, a magnetic device made of a magnet, a PTFE septum and a magnetic nanocomposite (Fe_3_O_4_@C18) is employed for the microextraction. Wang et al. [99] determined five parabens and TCS in biological samples, using a magnetic μSPE chip connected directly with the chromatographic system. Fresco-Cala and Cárdenas [100] synthetized a composite based on carbon nanohorns inside pipette tips for the determination of a group of four parabens in urine. Wang et al. [101] developed a device with GO packed into polyamide organic membrane for the analysis of different parabens in water, and finally, Montesdeoca-Esponda et al. [102] analysed benzotriazole UV stabilizers in sewage water by fabric phase sorptive extraction (FPSE), with a PDMS nanocomposite bonded on a polystyrene support as extraction device. 

**Figure 2:**
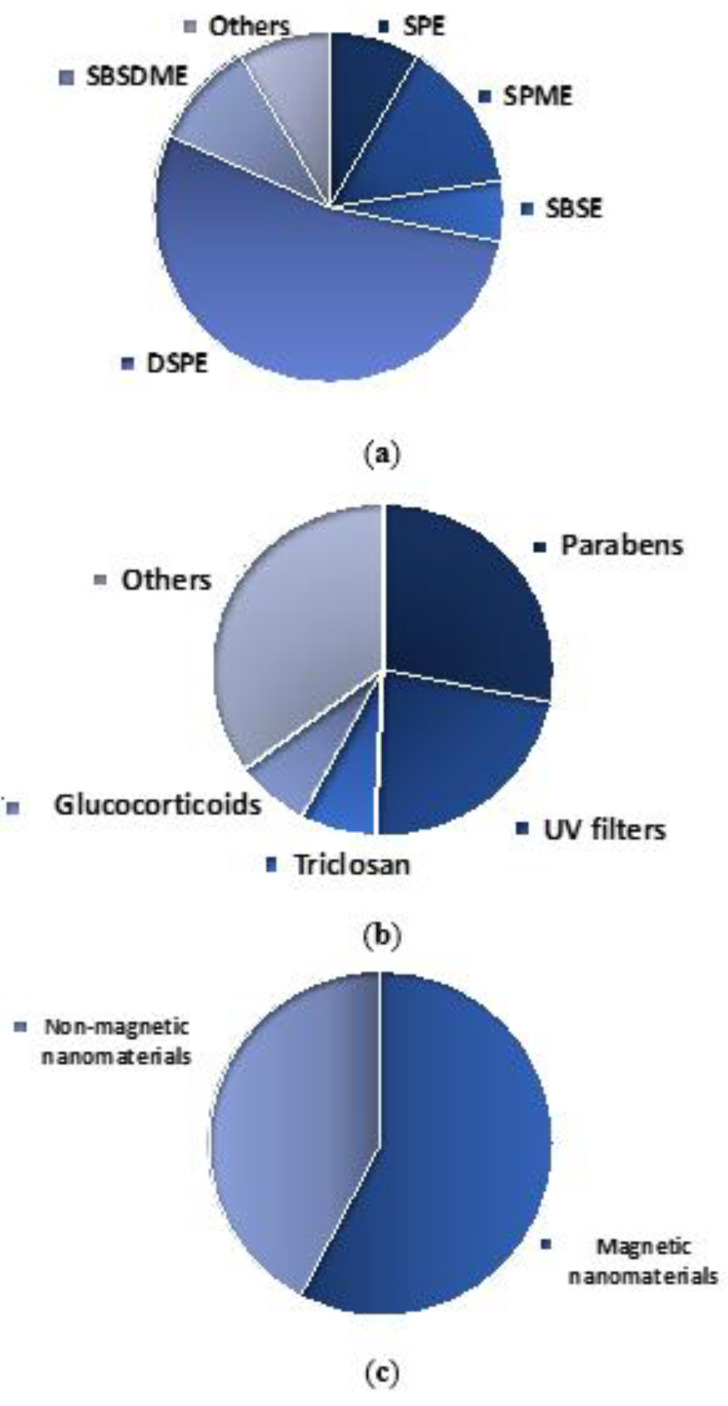
(**a**) Distribution of nanomaterials-based (micro)extraction techniques used in the determination of cosmetic-related compounds. SPE (solid-phase extraction); SPME (solid-phase microextraction); SBSE (stir bar sorptive extraction); DSPE (dispersive solid-phase extraction); SBSDME (stir bar sorptive dispersive microextraction). (**b**) Distribution of analytes studied. (**c**) Distribution of the materials employed.

As summary, we would like to emphasize that, regarding to the nanomaterials-based microextraction techniques used for the determination of cosmetic-related compounds, those based on the dispersion of the sorbent (i.e., DSPE and SBSDME) represent more than half of the published articles, as it is shown in Figure 2a. As commented before, the reduction of the extraction time, most probably, is the reason behind this trend.

With regard to the target analytes, authors paid attention during many years to the determination of parabens and UV filters, which gather about half of the published articles, as it is shown in Figure 2b.

Finally, with regard to the use of nonmagnetic or magnetic materials, Figure 2c shows that they are practically on par, but the observed trend is an increase in the use of magnetic materials in the last years.

## 4. Conclusions and Future Trends

In the last years, new analytical methods have been developed in order to control the presence of nonintended prohibited compounds in cosmetics products. Moreover, the presence of cosmetic ingredients and/or their metabolites in biological and environmental samples has also been studied. After an exhaustive revision of these methods, a clear trend in the use of nanomaterials for the determination of these cosmetic-related compounds has been observed, in line with the general trend observed within the analytical chemistry field. The high surface area, in addition to the thermal and chemical stability, and the easy fabrication/functionalization, make nanomaterials as excellent sorbents for any matrix.

In this review, the evolution of the impact of the use of nanomaterials for the extraction of cosmetic-related compounds has been studied. It should be noted that in the early 2010s (i.e., from 2010 to 2013), only eleven articles about this topic were published. By contrast, only in this last year, more than 10 articles were published, proving the high interest in this issue. 

Moreover, focusing on the type of microextraction technique, DSPE has been highly employed reaching more than half of the reported articles. Its simplicity and low cost compared with other well-stablished techniques such as SPME or SBSE, in addition to the possibility of reducing the total analysis time employing magnetic nanomaterials, have increased its popularity in recent years, and its use with novel magnetic materials is a clear trend. It should be noticed that 42 of the 72 articles reported in this review employ novel magnetic sorbents to achieve the extraction.

Regarding the target analytes, the major research of the published articles is performed on the extraction of parabens and/or UV filters, either in cosmetics, biological or environmental samples. These analytes are probably the most controversial ingredients in cosmetic products, along with potentially allergenic perfumes, and this might be the reason for the attention that has been given to them. However, in our opinion, other compounds have received less attention from the analytical chemistry community. We are referring to all those compounds not allowed in cosmetics due to its harmful effects, but that could be present at trace level due to unintended causes, e.g., impurities from raw materials, degradation of some ingredients, migration from the containers, cross reactions between ingredients, etc. For this reason, it is necessary to focus our attention on them, e.g., N-nitrosamines and polycyclic aromatic hydrocarbons, among others. Fortunately, there are already a few incipient efforts in this regard, but in our opinion, more efforts should be performed and most probably, it will be established as one of the future trends in this field. With regard to biological matrices, it is difficult to predict a future trend, since the cosmetic industry is continuously innovating cosmetic ingredients, but it is sure that both percutaneous absorption and metabolism studies should be conducted on new ingredients. Finally, from an environmental surveillance point of view, the researchers should focus their attention to all those cosmetic ingredients that easily reach the environment and cause a negative impact on flora and fauna. So, besides UV filters, which have been extensively studied, preservatives other than parabens (which are being less used due to the bad opinion from the consumers and the recent prohibition on some of them) could constitute a good choice. 

## Figures and Tables

**Table 1:** Published papers on cosmetic-related compounds determination by nanomaterials-based solid phase extraction.

Analyte(s) ^a^	Matrix	Extraction Technique ^b^	Material/Composite ^c^	Instrumental Technique ^d^	LOD (ng L^−1^)	RSD (%)	RR (%)	Year	Ref.
Parabens	Cosmetic	SPE	MWCNT	C-CAD	500–2100	<7.6	96–104	2010	[28]
GCCs	Cosmetic	SPE	MWCNT-MIP	LC-UV	5000	<2.1	83–106	2010	[30]
p-aminobenzoic acid	Cosmetic	SPE	NI-Zn-LDH	UV	3780	1.2	96–101	2014	[33]
Sulphonamides	Cosmetic	SPE	GO-PVC	LC-UV	3400–7100	<7.6	88–102	2015	[32]
Benzotriazole UV stabilizers	Cosmetic and environmental	SPE	GO	LC-UV	20–80	<8.1	89–105	2018	[29]
BPA	Cosmetic	SPE	SiO_2_@MIP	LC-FLD	229	<9	87–97	2018	[31]

^a^ BPA: bisphenol A; GCCs: glucocorticoids. ^b^ SPE: solid-phase extraction. ^c^ GO: graphene oxide; LDH: layered double hydroxides; MIP: molecularly imprinted polymer; MWCNT: multiwalled carbon nanotube; PVC: polyvinyl chloride. ^d^ C-CAD: corona-charge aerosol detector; FLD: fluorescence detector; LC: liquid chromatography; UV: ultraviolet detector.

**Table 2:** Published papers on cosmetic-related compounds determination by nanomaterials-based solid phase microextraction.

Analyte(s) ^a^	Matrix	Extraction Technique ^b^	Material/Composite ^c^	Instrumental Technique ^d^	LOD (ng L^−1^)	RSD (%)	RR (%)	Year	Ref.
GCCs	Cosmetic	SPME	BMA-EDMA-rGO	LC-MS	130–1930	<14	84–104	2012	[43]
UV filters	Environmental	SPME	Ti-TiO_2_/ZrO2	LC-UV	32–82	<11	77–114	2014	[38]
UV filters	Environmental	SPME	Co-S-AuNPs	LC-UV	25–56	<9.4	92–106	2014	[40]
Parabens	Cosmetic and environmental	SPME	SBA-15/PANI-p-TSA	GC-FID	80–400	<7	82–108	2015	[35]
UV filters	Environmental	SPME	Ph-TiO_2_-Ti	LC-UV	0.1–50	<9.1	86–106	2015	[39]
UV filters	Environmental	SPME	PANI/TiO_2_NTs/Ti	LC-UV	30–50	<7.7	86–113	2017	[37]
UV filters	Environmental	SPME	PIL-MCC/MNPs	LC-UV	40–260	<10	71–119	2017	[41]
PAHs	Cosmetic	SPME	g-C_3_N_4_@rGO	GC-MS	1.0–2.0	<12	70–118	2017	[42]
Parabens	Environmental	SPME	PPY-AgNPs	LC-UV	10	<4.5	94–104	2018	[36]
TCS, BPA and CPs	Environmental	SPME	HAP@SiO_2_	LC-UV	12–14	<8.2	90–110	2018	[44]

^a^ BPA: bisphenol A; CPs: chlorophenols; GCCs: glucocorticoids; PAHs: polycyclic aromatic hydrocarbons, TCS: triclosan. ^b^ SPME: solid-phase microextraction. ^c^ AP: aminopropyl; BMA: butyl methacrylate; EDMA: ethylene dimethacrylate; g-C_3_N_4_: graphitic carbon nitride; MCC: monolithic capillary column; MNPs: magnetic nanoparticles; NPs: nanoparticles; NTs: nanotubes; PANI: polyaniline; Ph: phenyl; PIL: polymeric ionic liquid; PPY: polypyrrole; rGO: reduced graphene oxide; SBA-15: mesoporous silica nanoparticles; TSA: toluene sulphonic acid. ^d^ FID: flame ionization detector; GC: gas chromatography; LC: liquid chromatography; MS: mass spectrometry detector; UV: ultraviolet detector.

**Table 3:** Published papers on cosmetic-related compounds determination by nanomaterials-based stir bar sorptive extraction.

Analyte(s) ^a^	Matrix	Extraction Technique ^b^	Material/Composite ^c^	Instrumental Technique ^d^	LOD (ng L^−1^)	RSD (%)	RR (%)	Year	Ref.
Parabens	Cosmetic and biological	SBSE	MIL-68	LC-MS/MS	1–2	<9.7	73–104	2018	[46]
UV filters	Environmental	SBSE	CNH/MA	LC-UV	100–1000	<7.9	71–124	2018	[47]
MI, BHT, BHA	Cosmetic	SBSE	GO-PEG-PANNL	GC-MS	500–5000	<3	84–107	2018	[48]
CPs	Cosmetic	SBSE	Fe_3_O_4_-rGO/g-C_3_N_4_	LC-UV	200–300 ng kg^−1^	<12	85–104	2018	[49]

^a^ BHA: butylated hydroxyanisole; BHT: butylated hydroxytoluene; CPs: chlorophenols; MI: 2-methyl-3-isothiazolinone. ^b^ SBSE: stir bar sorptive extraction. ^c^ CNH: carbon nanohorns; GO: graphene oxide; g-C_3_N_4_: graphitic carbon nitride; MA: methacrylate; PANNL: natural latex; PEG: polyethylene glycol; rGO: reduced graphene oxide. ^d^ GC: gas chromatography; LC: liquid chromatography; MS: mass spectrometry detector; UV: ultraviolet detector.

**Table 4:** Published papers on cosmetic-related compounds determination by nanomaterials-based dispersive solid phase extraction.

Analyte(s) ^a^	Matrix	Extraction Technique ^b^	Material/Composite ^c^	Instrumental Technique ^d^	LOD (ng L^−1^) ^e^	RSD (%)	RR (%)	Year	Ref.
TCS	Environmental	DSPE	MWCNT@MIP	LC-UV	n.r.	<12	91–95	2010	[54]
UV filters	Environmental	(M) DSPE	CoFe_2_O_4_@oleic acid	GC-MS	0.2–6	<16	74–119	2011	[74]
Parabens	Environmental	(M) DSPE	Fe_3_O_4_@PANI	LC-UV	300–400	<2.4	86–109	2012	[57]
UV filters	Environmental	CP (M) DSPE	Fe_2_O_3_@C-PSx	LC-UV	1430–7500	<14.9	89–97	2012	[75]
Parabens	Environmental	(M) DSPE	Fe_3_O_4_-AP	GC-PID	50–300	<8	87–103	2013	[59]
Rhodamine B	Cosmetic and environmental	(M) DSPE	Fe_3_O_4_@PAN	Fl	100	<8.2	94–99	2013	[84]
Pb (II) Mn (II)	Cosmetic and biological	(M) DSPE	Fe_3_O_4_-MWCNTs	AA	600–1000	<4.3	n.r.	2013	[85]
Hormones	Cosmetic	DSPE	MIL-101(Cr)	LC-UV	360–910	<6.1	93–102	2014	[55]
Parabens	Cosmetic, biological and environmental	DSPE	HKUST-1	LC-UV	1500–2600	<15	57–101	2015	[51]
UV filters	Cosmetics	DSPE	MIL-101	LC-UV	900–1200	<10	94–105	2015	[53]
Parabens	Cosmetic	(M) DSPE	Fe_3_O_4_@PANI-rGO	GC-FID	1200–2800	<7.9	89–101	2015	[63]
Parabens	Cosmetic	(M) DSPE	Fe_3_O_4_-G-mSiO_2_-Ph	LC-UV	10,000–25,000	<5.61	79–106	2015	[64]
TCS and BPA	Environmental	(M) DSPE	Fe-Fe_2_O_3_/GO	LC-UV	80–100	<7.5	85–93	2015	[78]
TCS, BPA and CPs	Environmental	(M) DSPE	Fe_3_O_4_@PANI	LC-UV	100–130	<6.6	85–107	2015	[79]
Metronidazole	Cosmetic	(M) DSPE	Fe_3_O_4_@MIP	LC-UV	3000	<5.20	91–104	2015	[87]
Musks, phthalates and allergens	Environmental	(M) DSPE	Fe_3_O_4_-rGO-OCT	GC-MS	0.29–3.2	<9.4	83–105	2015	[88]
Parabens	Environmental	DSPE	GO-PANI	LC-UV	50–1800	<11.5	74–120	2016	[52]
Parabens and UV filters	Environmental	(M) DSPE	Fe_3_O_4_@MIM-PF6	LC-MS/MS	260–1350	<8.3	87–99	2016	[62]
Parabens	Cosmetic	(M) DSPE	Fe_3_O_4_@SiO_2_	GC-FID	200–900	<5.6	85–107	2016	[65]
TCS	Cosmetic	(M) DSPE	Fe_3_O_4_-MIL-100	LC-UV	30,000 ng Kg^−1^	<5.5	91–101	2016	[76]
Parabens	Environmental	(M) DSPE	CoFe_2_O_4_-PS	LC-MS	50–150	<8.5	81–105	2017	[58]
Parabens	Cosmetic and environmental	(M) DSPE	Fe_3_O_4_@βCD-BMIM-Cl	LC-UV	20–90	<14.9	80–117	2017	[68]
UV filters	Environmental	(M) DSPE	Fe_3_O_4_-GCB	LC-MS/MS	1–4	<15	81–115	2017	[72]
GCCs	Cosmetic	(M) DSPE	Fe_3_O_4_@dtMIP	LC-UV	15,000	<2.6	87–102	2017	[81]
Parabens	Cosmetic, biological and environmental	(M) DSPE	Fe_3_O_4_@COF	LC-UV	20	<4.9	86–102	2018	[67]
Parabens	Biological and environmental	(M) DSPE	Fe_3_O_4_-MWCNTs	GC-MS	30–2000	<9.2	81–119	2018	[69]
UV filters	Environmental	(M) DSPE	Fe_3_O_4_@PDA	LC-MS	60–130	<3	95–104	2018	[73]
GCCs	Cosmetic	(M) DSPE	Fe_3_O_4_@MIP	LC-UV	50,000	<2.7	94–98	2018	[80]
Whitening agents	Cosmetic	(M) DSPE	Fe_3_O_4_@COF	LC-FLD	0.1	<5.5	78–105	2018	[86]
Hg(II)	Cosmetic and environmental	DSPE	CDs	Fl	2800	<3.4	91–117	2019	[56]
Parabens	Environmental	(M) DSPE	Fe_3_O_4_@sylgard 309	LC-UV	20,000–30,000	<11.4	60–120	2019	[60]
Parabens	Environmental	(M) DSPE	Fe_3_O_4_@DC193C	LC-UV	2300–6300	<10.2	86–118	2019	[61]
Parabens and phthalates	Environmental	(M) DSPE	Fe_3_O_4_-MWCNTs-MIL-101	LC-UV	30–150	<7.5	38–71	2019	[66]
Parabens	Biological and environmental	(M) DSPE	γ-Fe_2_O_3_@HAP	GC-MS	5000–10,000	<4.2	95–106	2019	[70]
UV filters	Environmental	(M) DSPE	Fe3O4-1210 (Zr/Cu)	LC-UV	10–20	<3.6	88–114	2019	[71]
TCS and TCC	Biological	(M) DSPE	Fe_3_O_4_@COF	UPLC-MS/MS	5–20	n.r.	93–109	2019	[77]
GCCs	Cosmetic	(M) DSPE	Fe_3_O_4_-MIL-101/g-C_3_N_4_	UPLC-MS/MS	2	<5.5	77–113	2019	[82]
Rhodamine B	Cosmetic	(M) DSPE	γ-Fe_2_O_3_@imino-pyridine	Fl	1600	<2.7	91–97	2019	[83]
4,4′-thioaniline	Cosmetic	(M) DSPE	CoFe_2_O_4_@HNTs-Au-NPs	SERS	26,000	<10	72–104	2020	[89]

^a^ BPA: bisphenol A; CPs: chlorophenols; GCCs: glucocorticoids; TCC: triclocarban TCS: triclosan. ^b^ CP: cloud-point; DSPE: dispersive solid-phase extraction; (M): magnetic-based. ^c^ AP: aminopropyl; BMIM-Cl: 1-butyl-3-methylimidazolium chloride; βCD: β-cyclodextrin; CDs: carbon dots; COF: covalent organic framework; dtMIP: dual template MIP; GCB: graphitized carbon black; GO: graphene oxide; g-C_3_N_4_: graphitic carbon nitride; HAP: hydroxyapatite; HNT: halloysite nanotubes; LDH: layered double hydroxides; MIM-PF6: methylimidazolium hexafluorophosphate; MIP: molecularly imprinted polymer; mSiO_2_: mesoporous silica; MWCNT: multiwalled carbon nanotube; NPs: nanoparticles; PAN: poly(aniline-naphthylamide); PANI: polyaniline; PDA: polydopamine; Ph: phenyl; PS: polystyrene; PSx: polysiloxane; OCT: octylamine; rGO: reduced graphene oxide; TSA: toluene sulphonic acid. ^d^ AA: atomic absorption; FID: flame ionization detector; Fl: fluorimetry; FLD: fluorescence detector; GC: gas chromatography; LC: liquid chromatography; MS: mass spectrometry detector; SERS: surface-enhanced Raman scattering UPLC: ultraperformance liquid chromatography; UV: ultraviolet detector. ^e^ n.r.: not reported.

**Table 5:** Published papers on cosmetic-related compounds determination by nanomaterials-based stir bar sorptive dispersive microextraction.

Analyte(s) ^a^	Matrix	Extraction Technique ^b^	Material/Composite ^c^	Instrumental Technique ^d^	LOD (ng L^−1^)	RSD (%)	RR (%) ^e^	Year	Ref.
UV filters	Environmental	SBSDME	CoFe_2_O_4_@oleic acid	LC-UV	2400–30,600	<11	79–120	2014	[90]
UV filters	Environmental	SBSDME	CoFe_2_O_4_@oleic acid	LC-UV	1600–2900	<12	90–115	2016	[91]
UV filters	Environmental	SBSDME	CoFe_2_O_4_-nylon 6	TD-GC-MS	13–148	<11	0–116	2016	[93]
UV filters	Environmental	SBSDME	CoFe_2_O_4_@oleic acid	GC-MS	10–550 ng kg^−1^	<14	91–110	2019	[92]
TPP and DPP	Biological	SBSDME	CoFe_2_O_4_-Strata X-AW	LC-MS/MS	1.9–6.3	<8	81–111	2019	[94]
N-Nitrosamines	Cosmetic	SBSDME	CoFe_2_O_4_-MIL-101	LC-MS/MS	60–300	<13.9	96–109	2019	[95]
PAHs	Cosmetic	SBSDME	CoFe_2_O_4_-rGO	GC-MS	20–2500	<10	n.r.	2020	[96]

^a^ DPP: dipheny lphosphate; PAHs: polycyclic aromatic hydrocarbons, TPP: triphenyl phosphate. ^b^ SBSDME: stir bar sorptive dispersive microextraction; SBSE: stir bar sorptive extraction; SPE: solid-phase extraction; SPME: solid-phase microextraction. ^c^ AW: anion weak exchanger; rGO: reduced graphene oxide. ^d^ GC: gas chromatography; LC: liquid chromatography; MS: mass spectrometry detector; TD: thermal desorption UV: ultraviolet detector. ^e^ n.r.: not reported.

**Table 6:** Published papers on cosmetic-related compounds determination by other nanomaterials-based (micro)extraction techniques.

Analyte(s) ^a^	Matrix	Extraction Technique ^b^	Material/Composite ^c^	Instrumental Technique ^d^	LOD (ng L^−1^)	RSD (%)	RR (%)	Year	Ref.
Parabens	Environmental	MCE	Fe_3_O_4_-C18	GC-MS	23.2–86.1	<7.1	96–106	2013	[98]
Parabens	Environmental	μSPE	GO	GC-MS	5–10	<9.5	85–106	2014	[101]
Benzotriazole UV stabilizers	Environmental	FPSE	PDMS	UPLC-MS/MS	6.01–60.7	<29.2	35–99	2015	[102]
Parabens + TCS	Biological	Microflow injection	magnetic SPE PANI chip	LC-UV	1100–4500	<11	84–117	2017	[99]
Parabens	Cosmetic	Rotative SPME	MWCNTs-COOH	LC-UV	630–800	<5.8	83–103	2018	[97]
Parabens	Biological	DPX	CNH monolith	LC-UV	1000–7000	<16	80–116	2019	[100]

^a^ TCS: triclosan. ^b^ DPX: disposable pipette extraction; FPSE: fabric phase sorptive extraction; MCE: magnetically confined hydrophobic nanoparticles microextraction; μSPE: micro solid-phase extraction. ^c^ CNH: carbon nanohorns; GO: graphene oxide; MWCNT: multiwalled carbon nanotube; PANI: polyaniline; PDMS: polydimethylsiloxane. ^d^ GC: gas chromatography; LC: liquid chromatography; MS: mass spectrometry detector; UPLC: ultraperformance liquid chromatography; UV: ultraviolet detector.
